# Unveiling the Mechanistic Role of Lewis Acidic Ionic Liquids in Styrene Polymerization

**DOI:** 10.1021/acspolymersau.6c00024

**Published:** 2026-04-01

**Authors:** Thainá Araruna, Gabriel F. S. Brito, Gisele A. Medeiros, Gabriel V. S. Dutra, Fabricio Machado, Brenno A. D. Neto

**Affiliations:** † Laboratory of Chemical Processes Development, Chemistry Institute, University of Brasília, Campus Universitário Darcy Ribeiro, 70904-970 Brasília, DF, Brazil; ‡ Laboratory of Medicinal and Technological Chemistry, Chemistry Institute, University of Brasília, Campus Universitário Darcy Ribeiro, 70904-970 Brasília, DF, Brazil; § Molecular Sciences Graduate Program, Universidade Estadual de Goiás, 75132-400 Anápolis, GO, Brazil; ∥ Chemical Engineering Graduate Program, Federal University of Goiás, Campus Samambaia, 74690-900 Goiânia, GO, Brazil

**Keywords:** ionic liquids, Lewis acids, ESI-MS, mechanism, polystyrene

## Abstract

This work describes the use of a series of Lewis acids supported in different ionic liquids (ILs), based on an imidazolium cation, as catalysts for the cationic polymerization of styrene. The influence of these ILs, the effect of Lewis acids, temperature, catalyst concentration, and the presence of organic solvents were investigated to evaluate the best conditions for polymerization. These catalytic systems were effective to polymerize styrene in the temperature range of 50–90 °C, obtaining quantitative conversions in the presence of ILs in polymerization catalyzed by small amounts of Lewis acid. It was possible to synthesize polymers with wide ranges of number-average molar mass, in the range of 200,000–600 g mol^–1^, with a wide range of molar-mass dispersity, ranging from 1.43 to 4.67, and glass transition temperatures in the range of 40–71 °C by varying the catalytic system and/or the synthesis temperature. The polymerization mechanism was investigated by ESI-MS­(/MS) in positive mode with highly elucidative results. Tailored polystyrenes were successfully synthesized due to the fundamental properties of task-specific ILs, paving the way for the industrial application of catalyst systems based on ILs/Lewis acids.

## Graphical abstract

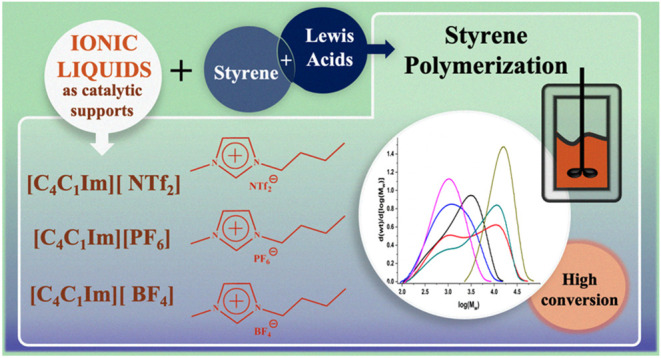

## 1 Introduction

Styrene is a versatile vinyl monomer that undergoes chain polymerization, including radical,^1^ anionic,^2^ and cationic^3^ mechanisms. This monomer has numerous applications in the plastics industry, particularly in the production of protective packaging, containers, and bottles. Polystyrene, in turn, is one of the most widely produced thermoplastics globally. However, the synthesis of polystyrenes, especially via ionic polymerization, typically requires large amounts of conventional organic solvents to stabilize the propagation species and to facilitate proper heat dissipation during the reaction. The main solvents used are mostly based on chlorinated compounds or toluene, which can cause serious harmful effects to health and the environment due to their toxic, volatile, and corrosive nature.

Cationic polymerization has enabled the preparation of a wide variety of advanced polymeric architectures and functional materials. Zhang et al.^4^ demonstrated that living cationic polymerization can be combined with polyhomologation to synthesize well-defined polyisobutylene–polyethylene block copolymers, which exhibited interesting properties for applications as crystallization modifiers for waxes and other self-assembled amorphous–crystalline polymeric systems. More recently, Eguchi et al.^5^ reported sequence-controlled cationic terpolymerization of styrene derivatives, oxiranes, and aromatic aldehydes, enabling the synthesis of high-molecular-weight terpolymers with defined monomer sequences. The resulting materials can be selectively degraded under acidic or oxidative conditions, opening possibilities for the development of responsive and recyclable polymeric systems. Despite the broad development of cationic polymerization, there are some disadvantages—for instance, the difficulty of separating the Lewis acid catalysts (e.g., BF_3_, AlCl_3_, AlBr_3_, TiCl_4_, SnCl_4_, AlCl_3_OBu_2_, and BF_3_OEt_2_) from the reaction products—which make reuse and disposal difficult.^6,7^ In order to minimize these disadvantages, the use of ionic liquids (ILs) as an alternative reaction medium stands out, due to its excellent physicochemical properties, such as negligible vapor pressure, good chemical and thermal stability, and ability to dissolve a wide range of organic, inorganic, and organometallic compounds, and their growing relevance in the context of green cationic polymerization has been highlighted in recent literature studies.^8–14^

In recent years, the use of ILs, especially those based on imidazolium cations, has been widely studied and successfully employed as solvents for a variety of reactions that typically exhibit low yields, poor selectivity, or other limitations when using traditional organic solvents.^15–22^ In the field of polymer science, ILs are used as components of polymeric matrices (e.g., additives, plasticizers, components of polymer electrolytes, and porogenic agents), as polymerizable ILs, catalysts, and, especially, as solvents. Due to their ionic nature, ILs are considered moderately polar solvents with a higher charge density, yet they are noncoordinating. This makes them ideal solvents for ionic polymerizations, as they allow for specific interactions between cationic or anionic species and growing polymer chains, potentially generating beneficial effects not observed with traditional organic solvents.^23–25^

Despite these advantages, studies on the application of ILs in cationic polymerizations remain limited. Rodrigues et al.^19^ synthesized IL catalysts based on imidazolium cation, incorporating iron atoms into their anionic structure. The authors demonstrated that the catalyst 1-butyl-3-methylimidazolium heptachlorodiferrate [C_4_C_1_Im]­[Fe_2_Cl_7_] exhibits high catalytic activity, achieving 71% conversion in just 15 min at 70 °C. However, under the same conditions, polymerizations conducted in the presence of ILs, 1-*n*-butyl-3-methylimidazolium bis­(trifluoromethylsulfonyl)­imide [C_4_C_1_Im]­[NTf_2_] and 1-*n*-butyl-3-methylimidazolium hexafluorophosphate [C_4_C_1_Im]­[PF_6_], yielded even higher conversions, due to the improved stability of the reaction medium provided by the ILs—for example, reaching 93% conversion in the presence of [C_4_C_1_Im]­[NTf_2_].

Wu et al.^26^ and Zhang et al.^27^ investigated the controlled cationic polymerization of isobutyl vinyl ether (IBVE) and *p*-methylstyrene using metallic co-initiators in the presence of ILs. The polymerizations proceeded in a milder exothermic manner, achieving higher conversions and yielding polymers with greater average molar mass compared with those carried out in conventional organic solvents.

Cationic polymerization of styrene has also been investigated in different reaction media, including supercritical carbon dioxide (scCO_2_), imidazolium-based ionic liquids such as [C_4_C_1_Im]­[PF_6_], and conventional organic solvents. In many of these systems, the resulting polymers typically exhibit relatively low molar masses. In contrast, reactions conducted in the presence of IL led to the formation of polystyrene with higher molar mass and conversion; additionally, the possibility of recovering and reusing IL imparts an eco-friendly feature to the polymerization system.^28,29^

Another recent study was reported by Mashayekhi et al.^30^ in which the initiator system for the cationic polymerization of 1-decene to form poly­(α-olefins) (PAO) was evaluated in the presence of three different ILs, whose cationic and anionic components were based on tributylamine (TBA), pyridine (Pyr), and triethanolamine (TEA). The authors compared the performance of the IL/AlCl_3_ catalyst system with that of AlCl_3_/H_2_O and reported superior performance for the system containing ILs. Pyr- and TBA-based ILs reduced the molar mass of PAO compared with PAO of pure AlCl_3_. The IL containing TEA had the opposite effect, increasing the molar mass. Furthermore, IL-initiated PAOs exhibited a higher long-chain branching content, which is advantageous for improving viscosity index properties.

Complementary results were reported by computational studies evaluating the cationic polymerization of isobutylene using the CumOH/BF_3_ system in [C_4_C_1_Im]­[NTf_2_], compared to CH_2_Cl_2_.^31^ The use of the IL led to lower activation barriers and enhanced stabilization of the cationic intermediates, resulting in higher molar masses and yields. The NTf_2_^–^ anion was identified as a key factor in controlling carbocation reactivity throughout the process.

Due to the impressive effects of ILs in different reaction systems,^32–36^ the present study was developed to provide insight into styrene polymerization carried out in the presence of ILs. We investigated the effect of different imidazolium ionic liquids [C_4_C_1_Im]­[NTf_2_], [C_4_C_1_Im]­[PF_6_], and [C_4_C_1_Im]·[BF_4_] on the behavior of styrene polymerization in the presence of various Lewis acids (InCl_3_, AlCl_3_, FeCl_3_, FeCl_2_, ZnCl_2_, CoCl_2_, CuCl_2_, SnCl_2_, MgCl_2_, BaCl_2_, CeCl_3_, ZnSO_4_, NiSO_4_, FeSO_4_, Nb_2_O_5_, CdO, Ni­(OAc)_2_, and MnO_2_). The effects of process variables such as reaction temperature and catalyst concentration on polymerization performance were also evaluated, and comparative experiments were conducted in polar and nonpolar aprotic organic solvents. Furthermore, high-resolution electrospray ionization mass spectrometry (ESI­(+)-MS) was employed to monitor InCl_3_-catalyzed styrene polymerization in the IL [C_4_C_1_Im]­[NTf_2_], allowing direct detection of transient ionic intermediates in real time. This mechanistic investigation provides molecular-level evidence of the interaction between the catalyst, monomer, and IL, revealing the formation of reactive chloronium species responsible for initiating and propagating the cationic polymerization pathway.

## 2 Experimental Methods

### 2.1 General

Reagents of analytical grade were purchased from commercial sources and used in the experimental part development without prior treatment. Liquid reagents and solvents were distilled prior to use and handled under moisture-minimizing conditions. Polymerizations were carried out in sealed tubes under an inert atmosphere to reduce the influence of adventitious water on the cationic process. InCl_3_ (99%), AlCl_3_ (99%), FeCl_3_ (97%), FeCl_2_ (98%), ZnCl_2_ (98%), CoCl_2_ (97%), CuCl_2_ (99%), SnCl_2_ (98%), MgCl_2_ (98%), BaCl_2_ (99%), CeCl_3_ (99%), NiSO_4_ (99%), Ni­(OAc)_2_ (98%), MnO_2_ (99%), Nb_2_O_5_ (99.9%), CdO (99.9%), 1-methylimidazole (99%), methanesulfonyl chloride (99.7%), acetone (99%), trifluoromethanesulfonic acid (98%), 1-chlorobutane (99%), ethyl acetate (99.5%), potassium tetrafluoroborate (96%), potassium hexafluorophosphate (99%), triethylamine (99%), styrene (99%), ZnSO_4_ (96%), alumina (97.5%), toluene (99.5%), FeSO_4_ (99%), *n*-butanol (99.4%), 1,4-dioxane (99%), dimethyl sulfoxide (99%), chloroform (99.9%), *N*,*N*-dimethylformamide (99.8%), acetonitrile (99.5%), magnesium sulfate (98%), sodium hydroxide (98%), and dichloromethane (99.5%) were used.

Nuclear magnetic resonance (^1^H NMR) spectra were obtained using an NMR spectrometer (Varian Mercury Plus, Varian Instruments) equipped with a 54 mm probe, operating at 600 MHz. Samples were prepared with deuterated chloroform (CDCl_3_) using tetramethylsilane (TMS) as the internal standard.

Thermal characterization of the samples was carried out by differential scanning calorimetry (DSC) using a Shimadzu DSC-60 calorimeter (Shimadzu Scientific Instruments). Initial sample masses were approximately 8.0 mg. The samples were cooled to −50 °C and then heated to 200 °C at a rate of 10 °C/min and under an inert helium atmosphere at a flow rate of 30 mL min^–1^. Data from the second heating cycle were used to determine the glass transition temperature (*T*_g_).

Mass spectrometry analyses (ESI-MS) were performed in the positive ionization mode over the *m*/*z* range of 150–700 using a quadrupole time-of-flight (Q-TOF) mass spectrometer (TripleTOF 5600+, AB Sciex) coupled to an ultrahigh-performance liquid chromatograph (UHPLC, Eksigent Ekspert 100-XL). The ion source was maintained at 500 °C with a capillary voltage of 5200 V. Samples were introduced by direct infusion using the flow injection analysis (FIA) technique. Data were acquired in information-dependent acquisition (IDA) mode to enable high-resolution detection of the most relevant ionic species formed during the reaction process. The reaction mechanism was monitored by mixing the catalyst and styrene in a 1:250 molar ratio in the presence of the IL [C_4_C_1_Im]­[NTf_2_]. A 10 μL aliquot of the reaction mixture was diluted in 1 mL of methanol. The resulting solution was directly injected into the ESI source immediately after mixing.

Average molar masses (and), molar-mass dispersity (*Đ*_M_), and molar-mass distributions (MMDs) of the polymer samples were determined by gel permeation chromatography (GPC) using a Viscotek GPCmax (Malvern Instruments Ltd.) equipped with a refractive index detector, a 200 μL injection loop, and three 300 × 8 mm^2^ separation columns (KF805L, KF804L, and KF802.5), installed in sequence, with stationary-phase maximum pore sizes ranging from 300 Å to 3 × 10^3^ Å. The calibration curve was constructed using standard polystyrene samples with average molar masses ranging from 1.2 × 10^3^ g mol^–1^ to 4.5 × 10^6^ g mol^–1^ and low molar-mass dispersities. Tetrahydrofuran (THF) was used as the mobile phase at 40 °C with a flow rate of 1 mL min^–1^. Prior to injection, sample solutions (2 mg of sample/2 mL of THF) were filtered through cellulose acetate membranes with 0.45 μm pore size.

### 2.2 Synthesis

#### 2.2.1 Synthesis of Ionic Liquids (ILs)

1-Butyl-3-methylimidazolium cation-based ILs, used as supports for the polymerization of styrene, were synthesized as described in the literature.^35,37^ Their chemical structures are listed in Figure 1.

**Figure 1:**
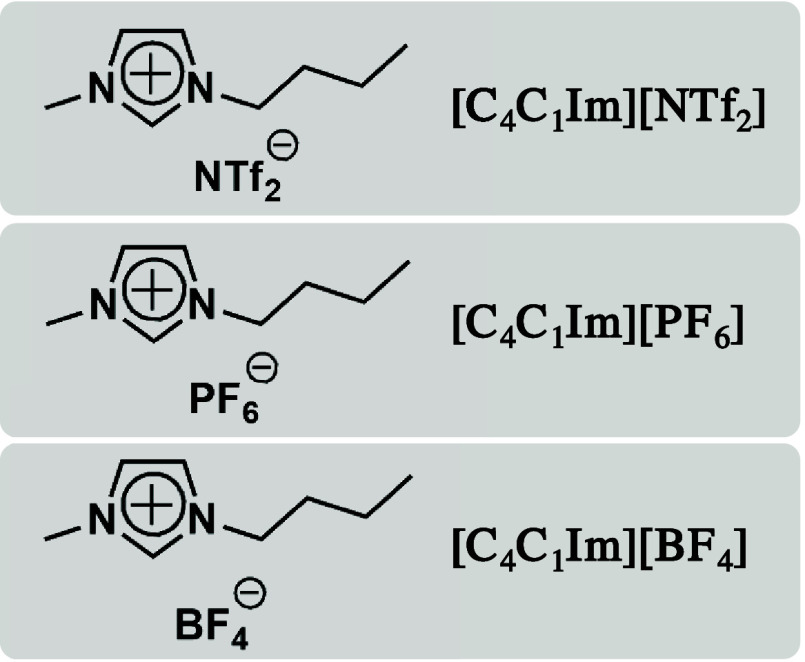
Representation of the chemical structures of ILs used as polymerization support.

**^1^H NMR (CDCl_3_, 600 MHz, δ in ppm)—[C_4_C_1_Im]­[NTf_2_]** (1-butyl-3-methylimidazolium trifluoromethanesulfonate): 8.67 (1H, s, NCHN), 7.44 (1H, m, CH_3_NCHCHN), 7.40 (1H, m, CH_3_NCHCHN), 4.20 (2H, t, *J* = 7.5 Hz, NCH_2_(CH_2_)_2_CH_3_), 3.94 (3H, s, NCH_3_), 1.88 (2H, m, NCH_2_ CH_2_CH_2_CH_3_), 1.39 (2H, m, N­(CH_2_)_2_CH_2_CH_3_), 0.96 (3H, t, *J* = 7.2 Hz, N­(CH_2_)_3_CH_3_).

**[C_4_C_1_Im]­[PF_6_]** (1-butyl-3-methylimidazolium hexafluorophosphate): 8.68 (1H, s, NCHN), 7.25 (1H, m, CH_3_NCHCHN), 7.10 (1H, m, CH_3_NCHCHN), 4.16 (2H, t, *J* = 7.4 Hz, NCH_2_(CH_2_)_2_CH_3_), 3.94 (3H, s, NCH_3_), 1.86 (2H, m, NCH_2_ CH_2_CH_2_CH_3_), 1.36 (2H, m, N­(CH_2_)_2_CH_2_CH_3_), 0.95 (3H, t, *J* = 7.3 Hz, N­(CH_2_)_3_CH_3_).

**[C**_**4**_ **C**_**1**_ **Im]­[BF**_**4**_ **]** (1-butyl-3-methylimidazolium tetrafluoroborate): 8.85 (1H, s, NCHN), 7.35 (1H, m, CH_3_NCHCHN), 7.28 (1H, m, CH_3_NCHCHN), 4.21 (2H, t, *J* = 7.4 Hz, NCH_2_(CH_2_)_2_CH_3_), 3.98 (3H, s, NCH_3_), 1.88 (2H, m, NCH_2_ CH_2_CH_2_CH_3_), 1.38 (2H, m, N­(CH_2_)_2_CH_2_CH_3_), 0.98 (3H, t, *J* = 7.3 Hz, N­(CH_2_)_3_CH_3_).

#### 2.2.2 Styrene Polymerizations

The polymerizations of styrene were carried out in the presence of different Lewis acids supported in the ILs (N.B.: ILs have the ability to form supramolecular aggregates arising from the combination of Lewis acid anions and imidazolium cations. Here, the expression “supported in the ILs” denotes a catalyst-in-IL system in which the ionic liquid serves as the supporting medium for the Lewis acids). An extensive library of Lewis acids was evaluated (InCl_3_, AlCl_3_, FeCl_3_, FeCl_2_, ZnCl_2_, CoCl_2_, CuCl_2_, SnCl_2_, MgCl_2_, BaCl_2_, CeCl_3_, ZnSO_4_, NiSO_4_, FeSO_4_, Nb_2_O_5_, CdO, Ni­(OAc)_2_, and MnO_2_) along with three different ILs [C_4_C_1_Im]­[NTf_2_], [C_4_C_1_Im]­[PF_6_], and [C_4_C_1_Im]­[BF_4_]. As a preliminary study, polymerizations were performed in sealed tubes containing 2.5 mL of styrene (purified by passing it through an activated aluminum oxide column to remove inhibitors prior to use) and 0.5 mL of IL, using a 1:1000 molar ratio of Lewis acid to monomer (i.e., 0.1 mol % catalyst), at 90 °C, under an inert atmosphere and magnetic stirring. Following previously reported procedures for similar reactions, after completion of reaction time, the mixture was cooled to room temperature and a small amount of dichloromethane (CH_2_Cl_2_) was added to stop the polymerization.^38,39^

In addition, polymerizations were conducted at different temperatures (ranging from 40 to 90 °C), at various Lewis acid concentrations (from 1 to 0.01 mol %), and in a range of polar and nonpolar aprotic organic solvents, including 1,4-dioxane, dimethyl sulfoxide, chloroform, toluene, dichloromethane, *N*,*N*-dimethylformamide, and acetonitrile.

## 3 Results and Discussion

Initially, we investigated the combination of different ILs acting as a support for the polymerization of styrene in the presence of various Lewis acids. The polymerizations were carried out using a 0.1 mol % catalyst concentration to promote a more efficient and sustainable process. Under the conditions studied in this work, the polymerization proceeded as a heterogeneous process due to the limited solubility of styrene in different ILs (for example, 1.0 g of [C4C_1_Im]­[PF_6_] dissolves about 0.7 g of styrene).^40^ The efficiency of Lewis acid catalysts in the presence of ILs has already been demonstrated by our group^41^ and others^42,43^ for different reactions.

Table 1 presents the behavior of different Lewis acids with respect to the conversion of styrene polymerization in the presence and absence of ILs. Initially, the reactions were carried out without fixing the polymerization time in order to evaluate the differences in reactivity among the transition-metal catalysts. Subsequently, additional reactions were conducted for 2 h to determine the most effective reaction system. Polymerizations performed in the absence of any Lewis acid resulted in only trace amounts of polymer, regardless of the ILs used, indicating that Lewis acids are responsible for initiating the polymerization.^44^

**Table 1:** Conversion of Styrene Polymerization in the Presence of Different ILs as a Reaction Support to Lewis Acid Catalysts^a^

	Lewis acid support			
Lewis acids	[C_4_C_1_Im][NTf_2_]	[C_4_C_1_Im][PF_6_]	[C_4_C_1_Im][BF_4_]	
InCl_3_	99%	10%	traces^b^	traces
	99% (2 h)	96% (118 h)	54% (118 h)	95% (2.5 h)
AlCl_3_	traces	traces	traces	traces
	10% (110 h)	81% (22 h)	traces (118 h)	68% (41 h)
FeCl_3_	92%	14%	traces	96%
	88% (2 h)	88% (41 h)	61% (95 h)	85% (13 h)
FeCl_2_	6%	12%	traces	92%
	61% (41 h)	85% (88 h)	traces (113 h)	89% (13 h)
ZnCl_2_	16%	7%	traces	traces
	81% (13 h)	87% (41 h)	traces (120 h)	61% (42 h)
CoCl_2_	traces	6%	traces	traces
	2% (115 h)	93% (67 h)	traces (120 h)	77% (64 h)
CuCl_2_	traces	traces	traces	traces
	2% (119 h)	63% (110 h)	traces (113 h)	traces (110 h)
SnCl_2_	traces	25%	traces	traces
	traces (118 h)	51% (44 h)	7% (118 h)	85% (110 h)
MgCl_2_	traces	traces	traces	traces
	47% (118 h)	traces (118 h)	19% (118 h)	72% (41 h)
BaCl_2_	traces	traces	traces	traces
	traces (119 h)	43% (64 h)	traces (119 h)	70% (110 h)
CeCl_3_	traces	4%	traces	traces
	traces (120 h)	92% (67 h)	traces (120 h)	75% (110 h)
ZnSO_4_	traces	traces	traces	traces
	65% (41 h)	67% (41 h)	traces (120 h)	75% (20 h)
NiSO_4_	traces	traces	traces	4%
	traces (120 h)	87% (87 h)	70% (86 h)	82% (45 h)
FeSO_4_	traces	traces	traces	traces
	traces (113 h)	88% (19 h)	traces (113 h)	75% (41 h)
Nb_2_O_5_	1%	1%	traces	3%
	63% (44 h)	85% (44 h)	44% (118 h)	76% (20 h)
CdO	traces	traces	traces	traces
	66% (49 h)	74% (49 h)	traces (120 h)	71% (118 h)
Ni(OAc)_2_	traces	traces	traces	traces
	4% (87 h)	51% (87 h)	traces (120 h)	67% (45 h)
MnO_2_	traces	traces	traces	traces
	traces (120 h)	61% (67 h)	traces (120 h)	71% (118 h)

a Reaction conditions: Concentration of 0.1 mol % of Lewis acids at 90 °C. Yields after 2 h and at different times, with the numbers in parentheses representing the polymerization time. In the absence of IL, the reaction volume was adjusted to maintain the same styrene concentration.

b Traces: ≤1% yield.

All Lewis acids, in combination with different ILs, were able to catalyze the polymerization of styrene at 90 °C. Some systems showed high conversions within a few hours of reaction—for instance, FeCl_3_ and InCl_3_ supported in [C_4_C_1_Im]­[NTf_2_]. Others, however, required 19–87 h of reaction to obtain polystyrene with high conversions, as in the cases of FeSO_4_, AlCl_3_, CoCl_2_, and NiSO_4_ supported in [C_4_C_1_Im]­[PF_6_]. The conversions achieved with this latter group of Lewis acids are comparable to those reported by Biedroń and Kubisa,^40,45,46^ who studied the cationic polymerization of styrene, at room temperature and under high temperatures, using alkyl chlorides/TiCl_4_ catalysts supported in [C_4_C_1_Im]­[PF_6_]. In contrast, the catalytic systems investigated here do not require high concentrations of either the catalyst or the IL. Bueno et al.^28^ also polymerized styrene cationically in the presence of [C_4_C_1_Im]­[PF_6_], achieving high conversions at 25 °C and 2 h of synthesis. However, they used high concentrations of AlCl_3_ (5.8 mol %), corresponding to a molar ratio nearly 60 times higher than that employed in the present study. Therefore, the results reported here highlight the potential of Lewis acids supported in different ILs as catalysts that are as effective, or even more effective, than those described in the previous literature.

In general, higher conversions were obtained using the Lewis acids supported in [C_4_C_1_Im]­[PF_6_], for example, in the presence of FeSO_4_, CuCl_2_, SnCl_2_, CoCl_2_, AlCl_3_, CeCl_3_, CdO, and MnO_2_. This behavior can be attributed to the beneficial effect of temperature on the solubility of styrene in this particular IL, which favors the efficiency of polymerization reactions due to the stabilization of the propagation species of the polymeric chain through ion-pairing effects.^47^

The difference in the reactivity of Lewis acids within the same reaction medium is explained by two factors:^7^ (i) hard and soft acid–base principle: briefly, reactions tend to occur more readily between hard acids and hard bases, or between soft acids and soft bases. This explains the greater efficiency of polymerization in the presence of the FeCl_3_, InCl_3_, and AlCl_3_ catalysts compared to FeCl_2_, ZnCl_2_, CoCl_2_, CuCl_2_, and SnCl_2_, as the former are classified as hard acids, whereas the latter are considered soft acids; and (ii) the strength of the interaction between the Lewis acid and the chloronium cation (which can be seen in the mechanism illustrated in Scheme 1) of the chain propagation species.^19^

**Scheme 1:**
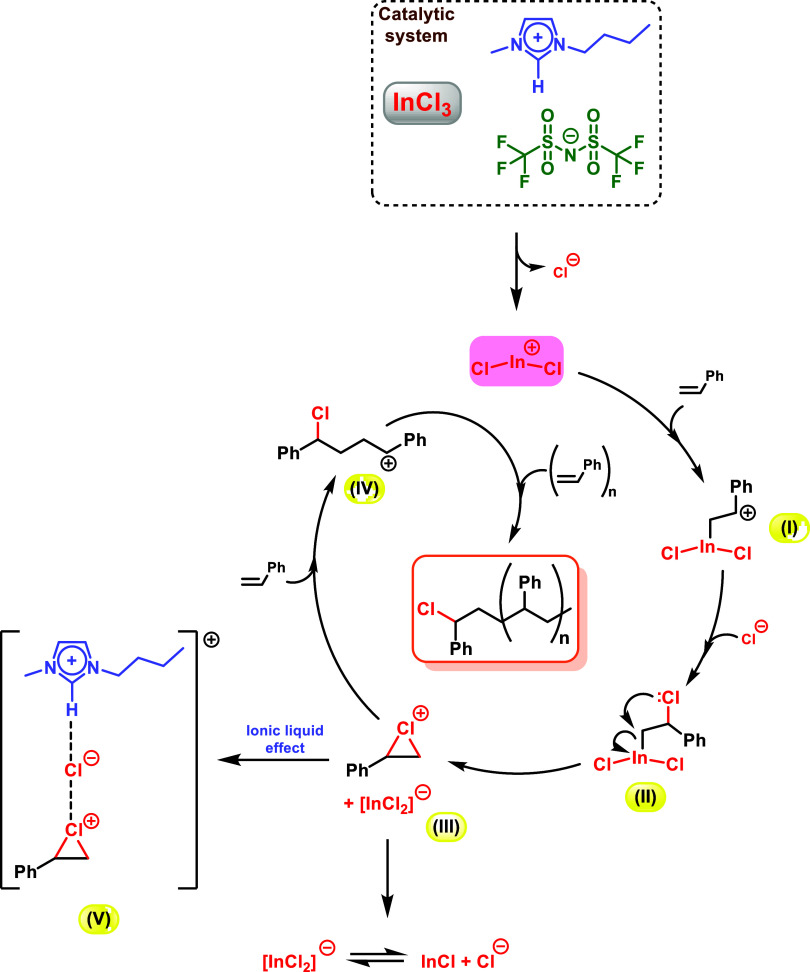
Proposed Mechanism, Based on ESI­(+)-MS­(/MS) Data, for the Cationic Polymerization of Styrene Catalyzed by InCl_3_ in the IL [C_4_C_1_Im]­[NTf_2_]

Indium-based catalysts provided the best results, producing polystyrene with 99% conversion when supported in [C_4_C_1_Im]­[NTf_2_]. For this reason, the catalytic system based on InCl_3_ supported in [C_4_C_1_Im]­[NTf_2_] was selected, and the polymerization behavior at different synthesis temperatures, ranging from 40 to 90 °C, was evaluated. The conversions obtained under these conditions are listed in Table 2. The higher the synthesis temperature, the greater the styrene conversion. At temperatures above 50 °C, the system becomes so reactive that it leads to polystyrene formation in nearly quantitative yields. This behavior is attributed to the increased reactivity of the catalytic species at higher temperatures, which enhances the propagation rate. In contrast, at lower temperatures, the catalytic species are less reactive, resulting in reduced conversion.

**Table 2:** Effect of Temperature on the Conversion of Styrene Polymerization Using InCl_3_ Supported in [C_4_C_1_Im]­[NTf_2_]­^a^

entry	temperature (°C)	conversion (%)
1	40	6
2	50	99
3	60	97
4	70	98
5	80	98
6	90	99

a Reaction conditions: Concentration of 0.1 mol % of InCl_3_ and 2 h of synthesis.

Since quantitative conversion was achieved at 50 °C and the use of low concentrations of Lewis acids is desirable to obtain a more economical and sustainable process, the effect of the catalyst concentration on styrene polymerization was studied at this temperature. Polymerizations were carried out using different concentrations of InCl_3_ catalyst supported in [C_4_C_1_Im]­[NTf_2_], varying down to 0.01 mol %. The conversions obtained under these conditions are given in Table 3. The results are very promising, especially considering that cationic polymerization with quantitative conversion was successfully achieved using only 0.1 mol % of catalyst in 2 h at 50 °C. A further decrease in catalyst concentration (below 0.1 mol %) led to lower conversions due to a significant reduction in the availability of active catalytic species.

**Table 3:** Effect of InCl_3_ Amount on Styrene Conversion in Polymerizations at 50 °C Using [C_4_C_1_Im]­[NTf_2_] as the Catalyst Support^a^

entry	InCl_3_ (mol %)	conversion (%)
1	1.00	99
2	0.20	99
3	0.10	99
4	0.09	2.6
5	0.08	5.3
6	0.07	2.2
7	0.06	traces^b^
8	0.05	traces
9	0.04	traces
10	0.03	traces
11	0.02	traces
12	0.01	traces

a Reaction conditions: Temperature of 50 °C for 2 h.

b Traces: ≤1% yield.

The combination of InCl_3_ in very small amounts with [C_4_C_1_Im]­[NTf_2_] as a catalyst support opens new avenues for exploring polymer formation via cationic polymerization. The combined effect of the IL and Lewis acid is very encouraging, mainly when compared to works published elsewhere.^25^ For instance, the group of Vijayaraghavan and MacFarlane^48–50^ used approximately 2.8–5.0 mol % of catalyst—bisoxalatoboric acid and its derivatives—to obtain polystyrene with conversions above 90% via cationic polymerization, conducted for 2 h at 60 °C in the presence of ILs. Verebélyi and Iván^51^ employed the initiation system 1-phenylethyl chloride/TiCl_4_ and benzotrifluoride, considered an environmentally benign solvent, and obtained polystyrene with high conversions (above 80%) in just 5 min at temperatures ranging from −20 to 25 °C. However, they used very high catalyst concentrations—around 53.3 mol %.

The evolution of conversion and average molar masses over time were also investigated, and the corresponding profile is shown in Figure 2. It was observed that the polymerization exhibited a low conversion rate up to 30 min, after which a 99% conversion was achieved in just 40 min of reaction. This abrupt increase in conversion is attributed to the rise in the medium’s viscosity, triggering kinetic phenomenon that can be described as the “cationic gel effect”.

**Figure 2:**
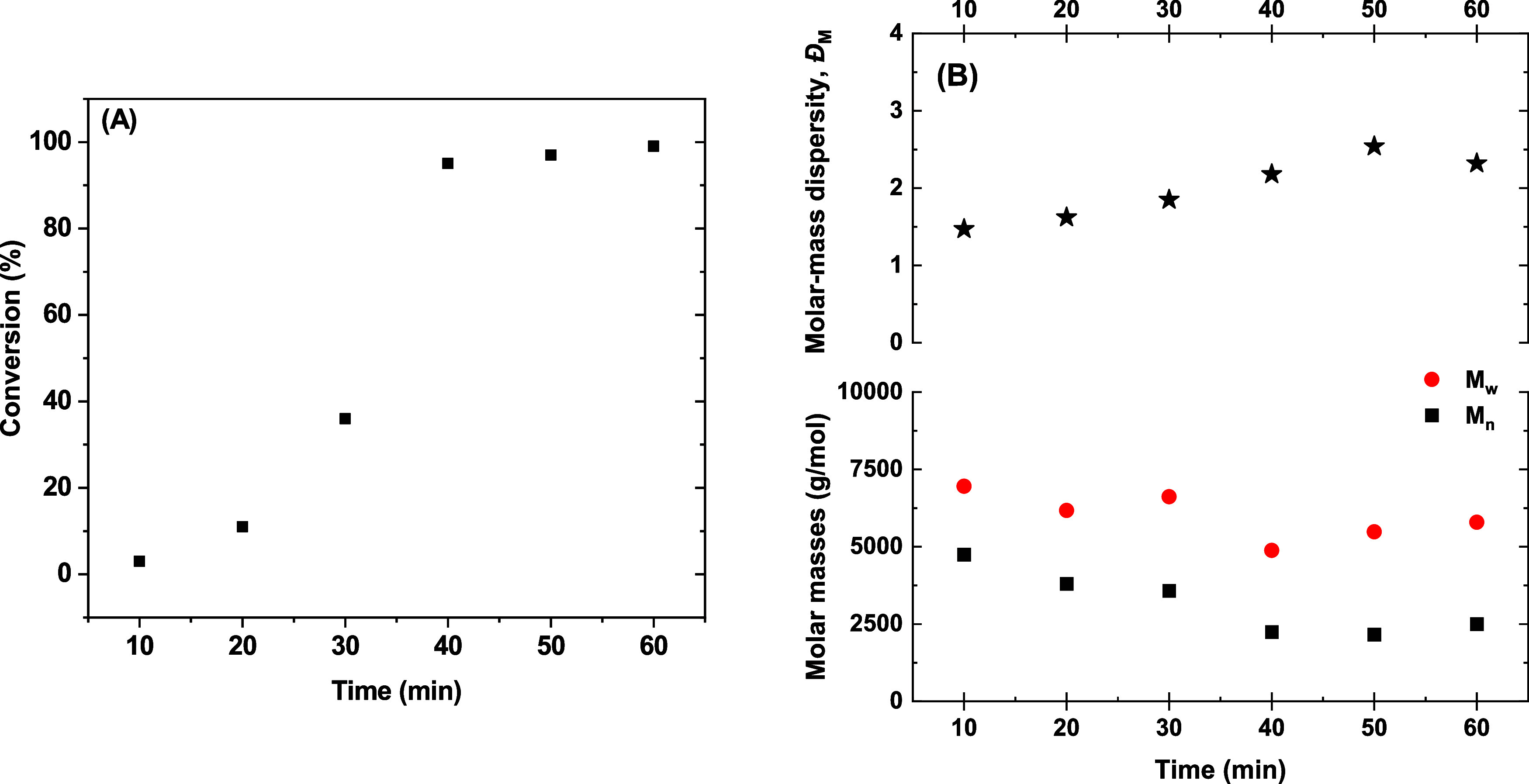
(A) Conversion of the polymerization of styrene using InCl_3_ supported in [C_4_C_1_Im]­[NTf_2_] as a function of polymerization time and (B) average molar masses and molar-mass dispersity profiles. Reaction conditions: Concentration of 0.1 mol % of InCl_3_ and temperature of 50 °C.

The gel effect, originally developed for radical polymerizations and extensively documented in radical systems, is associated with the reduced mobility of growing polymer chains, which leads to a decrease in the termination rate and, consequently, self-acceleration of the polymerization rate. This phenomenon strongly influences the final properties of the polymeric material, resulting in increased molar-mass dispersity.^52^

As a matter of fact, the viscosity of the reaction medium can play a decisive role in determining the kinetics and mechanistic features of the cationic polymerization of vinyl monomers catalyzed by Lewis acids. Although viscosity effects remain less comprehensively characterized in Lewis-acid-catalyzed cationic polymerizations than in radical polymerizations, it is reasonable to assume that a cationic analogue of the Trommsdorff (gel) effect exists. However, its mechanistic origin differs fundamentally from that observed in radical polymerization, as viscosity effects manifest distinctively in cationic systems.^53,54^

The autoacceleration behavior depicted in Figure 2A may depend on several factors, including the nature and concentration of the Lewis acid, monomer structure, reaction medium environment, and temperature, among others. Consequently, changes in medium viscosity, which affect how reactants diffuse through the medium, together with limitations in heat and mass transfer under diffusion-controlled kinetics, can strongly influence propagation rates, chain-transfer processes (to solvent, impurities, or counterions), ion-pair equilibria, the evolution of average molar masses, conversion profiles, and the potential for runaway exothermic reactions.

Experimental evidence shows that the medium viscosity strongly affects the final properties of polystyrene synthesized with using InCl_3_ supported in [C_4_C_1_Im]­[NTf_2_], resulting in increased molar-mass dispersity, as shown in Table 5 (entries 12–17), where the dispersity (*Đ*_M_) increases from 1.47 to 2.32 as the polymerization time increases from 10 to 40 min. According to Table 5 (entries 12–15), the number-average molar mass (*M̅*_n_) decreases from approximately 4700 to 2500 g mol^–1^ and the weight-average molar mass (*M̅*_w_) decreases from approximately 7000 to 5800 g mol^–1^, as polymerization time increases from 10 to 60 min.

The lowest temperature (50 °C) and the lowest catalyst molar percentage 0.1 mol % of InCl_3_ in the presence of [C_4_C_1_Im]­[NTf_2_] were identified as optimal conditions for obtaining polystyrene with quantitative conversions in 2 h of synthesis. Under these conditions, the effect of using polar and nonpolar organic solvents instead of ILs was evaluated, and the results are presented in Table 4. Very low conversions were obtained in polymerizations carried out in these organic solvents, with only trace amounts of polystyrene being detected.

**Table 4:** Effect of Organic Solvents on the Conversion of Styrene Polymerization Using InCl_3_ ^a^

entry	solvents	conversion (%)
1	*N*,*N*-dimethylformamide	0
2	dimethyl sulfoxide	traces
3	1,4-dioxane	traces
4	toluene	0
5	acetonitrile	0
6	chloroform	traces
7	dichloromethane	0

a Reaction conditions: 0.5 mL of solvent, concentration of 0.1 mol % of InCl_3_, and 2 h of synthesis at 50 °C.

These findings suggest that the Lewis acid catalyst was either unable to generate the propagation species of the polymer chain or that, if generated, it was stabilized by the solvent, leading to its deactivation. This latter effect was more pronounced with polar aprotic solvents. Regarding nonpolar aprotic solvents, the low conversions may be attributed to the sensitivity of cationic polymerizations to small concentrations of impurities. Overall, these results highlight the efficiency and promise of using ILs as an alternative medium for cationic polymerization of styrene, since no successful polymerization was achieved in conventional organic solvents.

The reaction mechanism of the developed catalytic cycle, [C_4_C_1_Im]­[NTf_2_]/InCl_3_, applied to the polymerization of styrene was investigated by high-resolution electrospray (tandem) mass spectrometry—ESI-MS­(/MS). We aimed to elucidate the IL effect^33^ and how the catalytic system acts in the efficient polymerization observed. This technique enabled molecular-level monitoring of the interactions between the Lewis catalyst (i.e., InCl_3_), the styrene monomer, and the IL [C_4_C_1_Im]­[NTf_2_], providing direct experimental evidence supporting a plausible reaction mechanism, as noticed in many reactions with transient ions.^55–59^ The reaction conditions were adapted to ensure an acceptable detection of representative ions for ESI­(+)-MS monitoring. The system was prepared using a 1:250 molar ratio (catalyst:monomer), which ensured an adequate concentration of the active species for MS detection. This adjustment was necessary because in previous experiments conducted at a 1:1000 ratio, the relevant and transient ionic signals showed low intensity, hindering the identification of intermediates formed during polymerization. Additionally, as soon as the reaction system was prepared, MS analysis was performed immediately to ensure the visualization of transient intermediates.

Figure 3A presents the ESI­(+)-MS spectrum obtained from the reaction mixture prior to the start of the polymerization heating, exhibiting a prominent signal of *m*/*z* 558.1672, attributed to the IL (2 cations, 1 anion) employed as the reaction medium, in accordance with the literature and the expected behavior of the IL [C_4_C_1_Im]­[NTf_2_].^60^ This finding indicates that the IL remains chemically intact within the system and effectively acts as both a solvent and stabilizing medium for the indium metal complexes formed in situ. It is known that indium is an excellent metal to trap carbenes formed from imidazolium-based ILs.^58^ Additionally, the high intensity of the signals related to the IL explains the difficulty in detecting other ions of interest, as a consequence of their strong signal suppression caused by the presence of IL supramolecular aggregates.^61–63^

**Figure 3:**
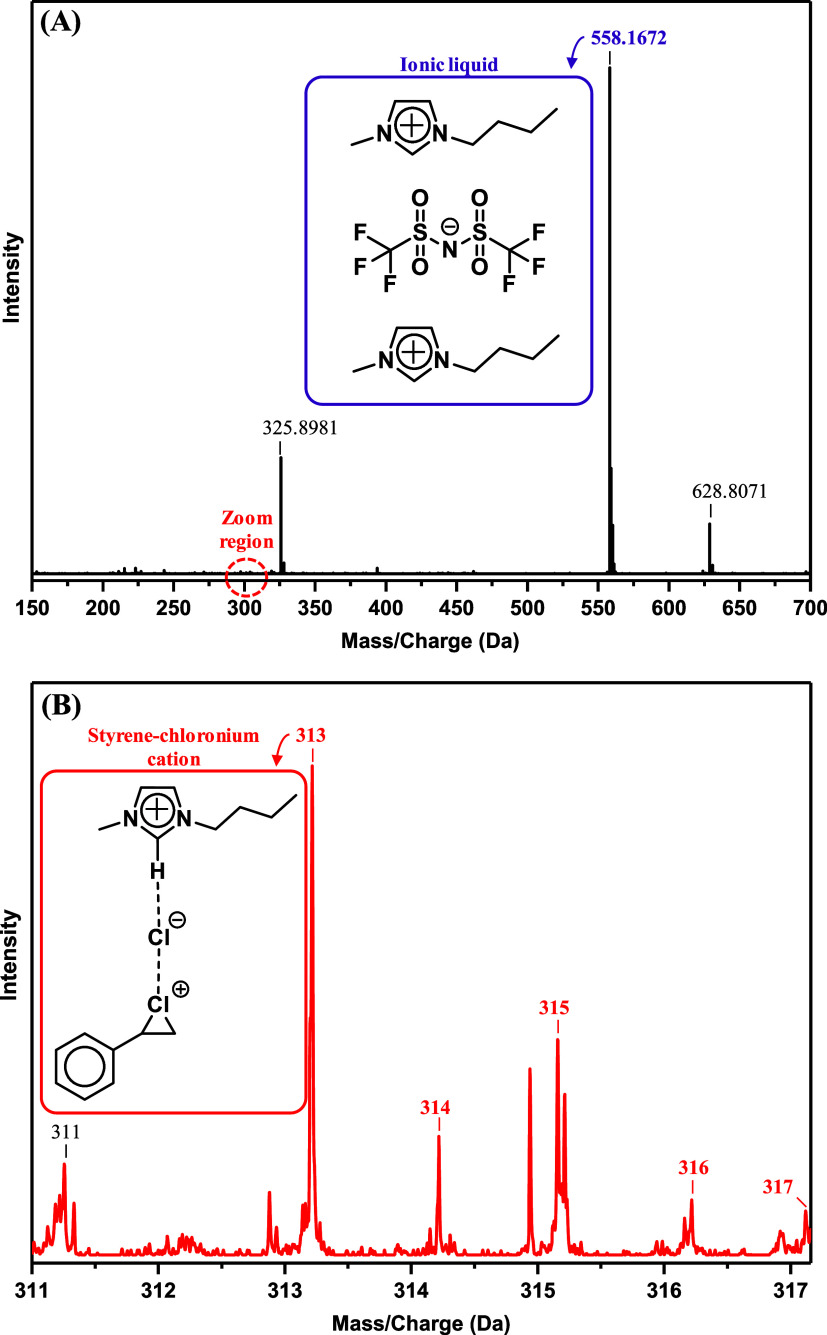
High-resolution spectra of (A) ESI­(+)-MS from *m*/*z* 150–700 of the reaction solution from the InCl_3_-catalyzed styrene polymerization in the IL [C_4_C_1_Im]­[NTf_2_] and (B) an ESI­(+)-MS expansion of the *m*/*z* 311–317 region, where the styrene–chloronium cation (*m*/*z* 313) is detected as a supramolecular species containing the IL [C_4_C_1_Im]­[NTf_2_].

Figure 3B enlarges the red-lighted region in Figure 3A, revealing the appearance of an ion of *m*/*z* 313. Its isotopic distribution (additional signals of *m*/*z* 314, 315, 316, and 317, highlighted in red) is consistent with the formation of the chloronium cation, whose proposed supramolecular structure is shown in Figure 3B, and reflects the expected ^35^Cl/^37^Cl pattern (two chlorine atoms), which is diagnostic of chlorine-containing species and supports this assignment. We note a small deviation in the exact mass and isotopologue distribution. However, at such low signal intensity, this is expected, as it is not possible to reliably determine the isotopologue distribution or clearly visualize the predicted signals. Therefore, this result requires caution in its interpretation and should be considered an underestimation. This species results from the interaction between indium chloride and the styrene double bond, representing the initial step of the InCl_3_-mediated cationic polymerization of styrene in the IL. The detection of this ion points to the electrophilic activation of the monomer in the reaction medium, indicating that polymerization is effectively initiated, similar to when iron-containing ILs are used as catalysts; however, in that case, the detection and characterization of this transient intermediate were unequivocal.^19^

The MS data simultaneously demonstrate both the stability and participation of the IL in the reactive phase as well as the generation of an active species responsible for polymer chain propagation. These findings validate the mechanism proposed for the InCl_3_/[C_4_C_1_Im]­[NTf_2_]/styrene system, illustrated in Scheme 1.

As depicted in Scheme 1, a complex mechanism takes place, although it is consistent with polymerization reactions previously described in ILs.^19,38,64,65^ Initially, InCl_3_ dissociates according to equilibrium InCl_3_ ⇌ [InCl_2_]^+^ [Cl]^−^. The cation [InCl_2_]^+^ is expected to act as a stronger Lewis acid and is responsible for activating the monomer. The cation [InCl_2_]^+^ is also considered the species responsible for the activation of the styrene double bond in our system, and this result is consistent with the findings reported by Rodrigues and co-workers, who identified [InCl_2_]^+^ as the catalytically active species in InCl_3_-mediated cycloaddition reactions.^66^ In their work, [InCl_2_]^+^ was shown to coordinate to unsaturated substrates, such as alkenes and alkynes, initiating cationic mechanisms.^66^ This supports the plausibility of [InCl_2_]^+^ activating the styrene double bond in our system.

Next, upon styrene coordination, **Int I** (Scheme 1) is formed. High-resolution MS indicates the interception of this transient intermediate, but its very low abundance, due to signal suppression by the IL, hinders its MS/MS characterization and isotopological validation. **Int I** undergoes intramolecular cyclization to afford **Int II** (Scheme 1), which in turn undergoes metal reduction, releasing the In^1+^ species and forming the chloronium ion **Int III** (Scheme 1).

The chloronium ion is capable of forming a supramolecular species of high interest, namely, **Int V** (detected and shown in Figure 3), which encompasses the chloronium ion, one chloride, and one imidazolium. This also highlights the importance of [NTf_2_]^−^, a nonnucleophilic anion with low coordination ability, which does not compete with the reactive chloride anion. The chloronium derivative then undergoes a second styrene addition, forming **Int IV** (Scheme 1), which proceeds with propagation of the polymerization reaction, as shown in Scheme 1. Overall, these results also explain why only a small amount of metal halide is required to perform this polymerization, since the initial formation of the chloronium ion is sufficient to trigger a highly efficient propagation reaction.

The average molar masses, molar-mass dispersity, and glass transition temperatures (*T*_g_) of selected polystyrene samples are presented in Table 5. These properties are highly dependent on the experimental conditions employed, such as the type and concentration of Lewis acid, the type and presence of IL, and the synthesis temperature. These variables led to variations in the number-average molar mass, ranging from 200,000 to 600 g mol^–1^, and the weight-average molar mass ranging from 426,000 to 1300 g mol^–1^. Dispersity ranged from low (1.43) to high (4.67), while *T*_g_ values varied from 71 to 40 °C.

**Table 5:** Average Molar Masses, Molar-Mass Dispersity, and Glass Transition Temperature (*T*_g_) of Polymers Synthesized with Different Lewis Acids^a^

entry	Lewis acids	ILs	*T* (°C)	time (min)	conversion (%)	(g mol^–1^)	(g mol^–1^)	*Đ*_M_	*T*_g_ (°C)
1	InCl_3_	[C_4_C_1_Im][BF_4_]	90	(118 h)	54	426,155	206,094	2.07	71.8
2	InCl_3_	[C_4_C_1_Im][PF_6_]	90	(118 h)	96	1415	692	2.05	25.0
3	InCl_3_	[C_4_C_1_Im][NTf_2_]	90	120	99	1922	1244	1.55	25.0
4	InCl_3_	[C_4_C_1_Im][NTf_2_]	80	120	98	3441	2230	1.54	25.8
5	InCl_3_	[C_4_C_1_Im][NTf_2_]	70	120	98	3854	2123	1.82	26.8
6	InCl_3_	[C_4_C_1_Im][NTf_2_]	60	120	97	4148	2292	1.81	28.4
7	InCl_3_	[C_4_C_1_Im][NTf_2_]	50	120	99	4407	2272	1.94	
8	InCl_3_	[C_4_C_1_Im][NTf_2_]	40	120	6	4351	2697	1.61	68.1
9^b^	InCl_3_	[C_4_C_1_Im][NTf_2_]	50	120	99	3805	1819	2.09	62.1
10^c^	InCl_3_	[C_4_C_1_Im][NTf_2_]	50	120	99	4494	2385	1.88	37.7
11^d^	InCl_3_	[C_4_C_1_Im][NTf_2_]	50	120	5.3	3975	2552	1.56	62.1
12	InCl_3_	[C_4_C_1_Im][NTf_2_]	50	10	2.8	6954	4746	1.47	71.4
13	InCl_3_	[C_4_C_1_Im][NTf_2_]	50	20	10.8	6174	3802	1.62	
14	InCl_3_	[C_4_C_1_Im][NTf_2_]	50	30	35.8	6617	3578	1.85	66.8
15	InCl_3_	[C_4_C_1_Im][NTf_2_]	50	40	95	4881	2238	2.18	40.4
16	InCl_3_	[C_4_C_1_Im][NTf_2_]	50	50	97	5483	2160	2.54	29.1
17	InCl_3_	[C_4_C_1_Im][NTf_2_]	50	60	99	5793	2496	2.32	33.1
18	FeCl_3_		90	120	96	6232	1336	4.67	
19	FeCl_3_	[C_4_C_1_Im][NTf_2_]	90	120	92	2702	1065	2.54	
20	FeCl_2_		90	120	92	7281	1677	4.34	51.4
21	FeCl_2_	[C_4_C_1_Im][PF_6_]	90	(88 h)	85	1797	777	2.31	38.8
22	ZnCl_2_	[C_4_C_1_Im][NTf_2_]	90	120	16	3855	2353	1.64	67.2
23	ZnCl_2_	[C_4_C_1_Im][NTf_2_]	90	(13 h)	81	3501	1714	2.04	
24	ZnCl_2_	[C_4_C_1_Im][PF_6_]	90	(41 h)	87	1332	660	2.02	31.8
25	CoCl_2_	[C_4_C_1_Im][PF_6_]	90	120	6	2492	1394	1.79	
26	CoCl_2_	[C_4_C_1_Im][PF_6_]	90	(63 h)	93	1345	707	1.90	30.3
27	SnCl_2_		90	(110 h)	85	16,063	11,280	1.42	25.0
28	SnCl_2_	[C_4_C_1_Im][PF_6_]	90	120	25	1278	731	1.75	40.1

a Reaction conditions: 0.1 mol % of catalyst.

b Amount of the Lewis acid: 1.0 mol %.

c Amount of the Lewis acid: 0.2 mol %.

d Amount of the Lewis acid: 0.08 mol %.

The average molar masses and dispersities obtained in this work encompass the full range reported in the literature for cationic polymerization of styrene in the presence of ILs, which typically yield polystyrene with between 11,000 to 650 g mol^–1^ and dispersities between 1.3 and 3.1.^28,40,45,46,48–50^ Thus, this study stands out by achieving high molar masses (Table 5, entry 1), polystyrenes with unimodal, bimodal, narrow, and broad distributions (Table 5, entries 18 and 20), and high conversions in a short reaction time while attaining properties comparable to those obtained in long synthesis periods^36^ (Table 5, entries 7, 9, and 15). The data compiled in Table 5 originate from different polymerization systems and experimental conditions and are therefore intended to illustrate the versatility of the catalytic platforms rather than a single directly comparable data set.

Polymerization reactions carried out with different Lewis acids (Table 5, entries 3, 19, and 22; and entries 2, 24, 25, and 28) demonstrate that the molar masses and the molar-mass dispersity of the resulting polystyrenes are significantly influenced by the nature of the catalyst (Figure 4). Table 5 (entries 7 and 9–11) shows that the concentration of Lewis acid has a relatively weak effect on the polymer properties, although a trend of increasing number-average molar mass is observed as the catalyst concentration decreases (Figure 4A). As the proportion of catalyst is reduced, fewer active sites are generated in the reaction medium, allowing a greater number of styrene molecules to be incorporated into each growing polymer chain. This results in the formation of polystyrenes with higher molar masses.^19^

**Figure 4:**
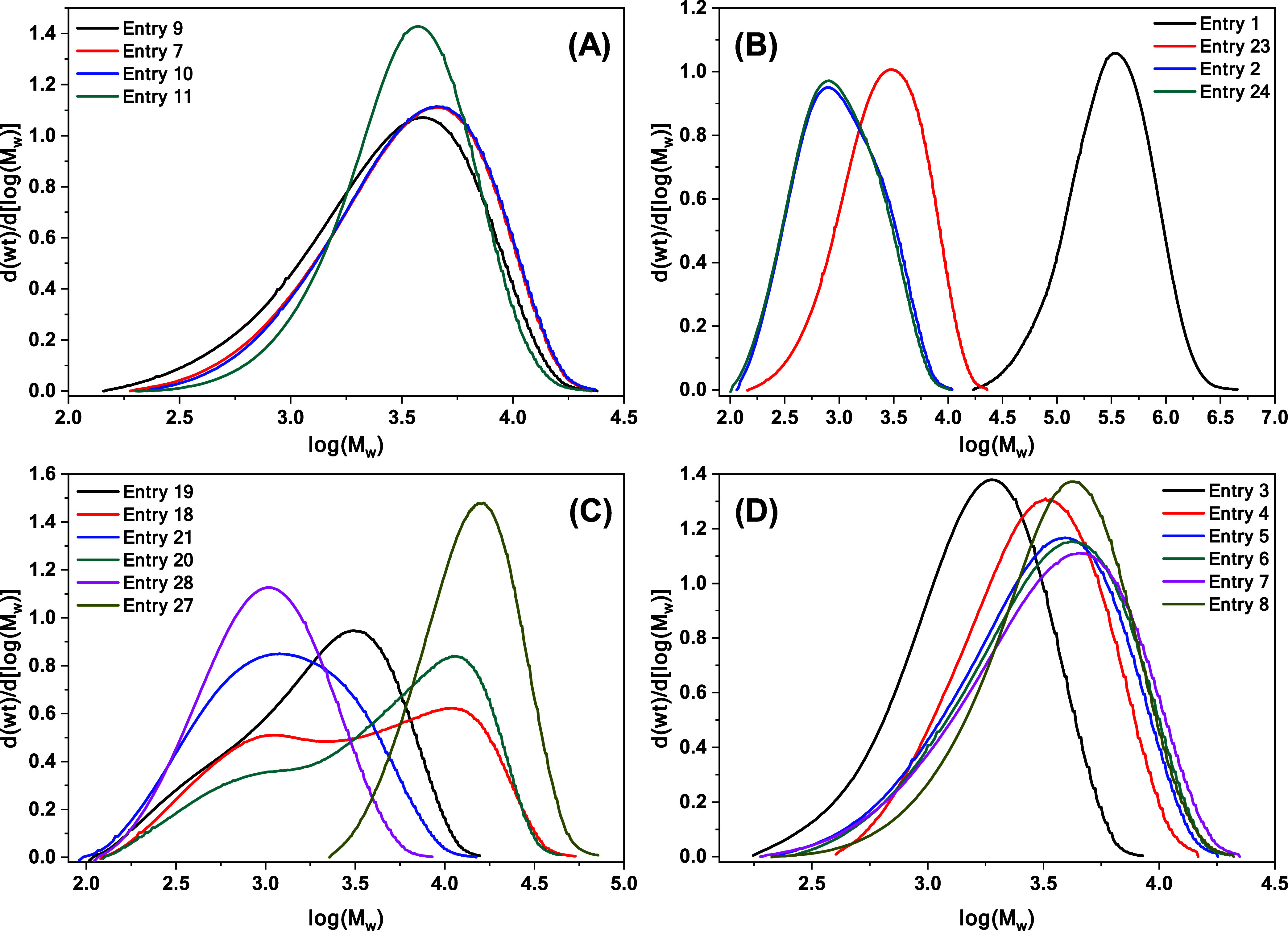
Molar-mass distribution curves: (A) effect of concentration of Lewis acid, (B) type of IL and (C) use of IL as a polymerization support, and (D) effect of the synthesis temperature. The entries shown in this figure correspond to the polymer samples listed in Table 5.

The type of IL used as a catalyst support also affects the polymer properties. Polystyrenes synthesized in less viscous ILs [C_4_C_1_Im]­[BF_4_] and [C_4_C_1_Im]­[NTf_2_] exhibited higher average molar masses (Table 5, entries 1 and 23) than those obtained, under the same conditions, using more viscous ILs [C_4_C_1_Im]­[PF_6_] (Table 5, entries 2 and 24). According to Zhang et al.,^67^ the high viscosity of ILs leads to a lower rate of heat dissipation from the reaction medium, which results in the formation of polymers with lower molar mass. Despite this, the type of IL employed did not significantly affect the molar-mass distribution curves of polystyrene, as shown in Figure 4B.

Regarding the use of ILs as catalyst supports, it was observed that polymerizations carried out in these ionic media (Table 5, entries 19, 21, and 28) produced polystyrenes with lower average molar mass and lower dispersity values compared to polymerizations performed in the absence of ILs (Table 5, entries 18, 20, and 27). This behavior is attributed to ion-pairing effects, which stabilize the propagating species of the polymer chain by facilitating charge displacement.^27^ The molar-mass distribution curves for these polystyrenes are shown in Figure 4C.

Polystyrenes with broader average molar masses and lower dispersities were obtained at a lower synthesis temperature (Table 5, entries 3–8), as shown in Figure 4D. This behavior is attributed to the fact that cationic polymerizations are better controlled at low temperatures, favoring the propagation mechanism of the polymer chains over the termination step and resulting in the formation of polymers with higher molar masses.^6,7^

The synthesized polystyrenes exhibited a wide range of molar-mass dispersities from 1.43 to 4.67. Such values are typically observed for polystyrene synthesized by radical, ionic, or the combination of both polymerization processes. However, the systems developed in this study do not require high synthesis temperatures or the use of organic solvents, making them more versatile, environmentally attractive, and economically viable.^19^

The *T*_g_ values of the samples can be correlated with the polymer molar mass, depending on the value range: polystyrenes with low molar mass tend to exhibit reduced *T*_g_ values (Table 5, entries 2 and 3), while samples with higher molar masses are associated with higher *T*_g_ values (Table 5, entries 1 and 12).^19^

## 4 Conclusions

The use of low concentrations of Lewis acids supported in ILs enabled the formation of polystyrene with high conversions at lower temperatures and in a shorter period of time than those typically reported in the literature. The polymeric properties were influenced by the type and concentration of the Lewis acid, the type and presence of the IL, and the synthesis temperature. The use of IL as a polymerization support proved especially promising for the preparation of polystyrenes with narrower molar-mass distribution. High-resolution ESI­(+)-MS analyses provided direct molecular-level evidence of the active ionic intermediates formed in the InCl_3_/[C_4_C_1_Im]­[NTf_2_]/styrene system, confirming the proposed cationic polymerization mechanism. The results indicate that applying ILs as a reaction media offers great potential for developing environmentally friendly polymerization systems to replace conventional organic solvents. This approach enables the synthesis of polymeric materials with distinctive properties that are often difficult to achieve in traditional solvent-based systems.
